# Metabolite Profiles of the Green Beans of Indonesian Arabica Coffee Varieties

**DOI:** 10.1155/2021/5782578

**Published:** 2021-11-23

**Authors:** Nizar Happyana, Amelinda Pratiwi, Euis Holisotan Hakim

**Affiliations:** ^1^Organic Chemistry Division, Faculty of Mathematics and Natural Sciences, Bandung Institute of Technology, Bandung, West Java, Indonesia; ^2^Department of Chemistry Education, Indonesia University of Education, Bandung, West Java, Indonesia

## Abstract

The green beans of 3 Indonesian arabica coffee varieties, namely, ateng, buhun, and sigararutang, were analyzed with ^1^H NMR-based metabolomics coupled with alpha-glucosidase inhibitory activity assay. These coffees were cultivated in the same geographical conditions. The PLSDA model successfully classified the green coffee beans based on their varieties. To reveal the characteristic metabolites for each coffee variety, S-plot of two-class OPLSDA models was generated and analyzed. Ateng coffee was characterized with trigonelline, sucrose, 5-CQA, and acetic acid. The characteristic metabolites of buhun coffee were citric acid and malic acid. Meanwhile, the most discriminant compound of sigararutang coffee was quinic acid. HCA analysis revealed the lineage relationship of the 3 coffee varieties. Ateng coffee had closer lineage relationship to sigararutang compared to the buhun coffee. Alpha-glucosidase inhibitory activity of the coffee samples did not differ widely. IC_50_ values of alpha-glucosidase inhibitory activity of ateng, sigararutang, and buhun coffees were 3.01 ± 0.16, 3.14 ± 0.20, and 5.05 ± 0.28 mg/mL, respectively. Although grown in the same geographical conditions, our results revealed that each coffee variety possessed a unique metabolome clarifying the diversity of Indonesian arabica coffees. This study verified that ^1^H NMR-based metabolomics is an excellence method for discovering the lineage relationship in the samples with different varieties or cultivars.

## 1. Introduction

*Coffea arabica* (arabica coffee) is the most cultivated coffee species in the world. Around 70% of available coffee in the markets worldwide is arabica coffee. This coffee is recognized possessing higher quality compared to other coffee species since it is rich in flavor, has less bitter, and contains low caffeine [1]. This makes arabica coffee as the most appreciated and consumed coffee beverages. The quality of arabica coffee is affected by several factors including the genetic characteristics of the cultivars, the environment condition, the agricultural management, and the postharvest processing [2]. The first two are considered as the most determining factors of the coffee quality [3]. The artificial and natural cross breeding have been applied as the common methods for obtaining the best genetic cultivars that able to adapt to the specific environmental conditions, resist to pests and diseases, and possess a high productivity and a better flavor [4].

As one of the largest coffee producers and exporters in the world, Indonesia has many superior arabica coffee varieties, including ateng, buhun, and sigararutang. Ateng and sigararutang coffees are extensively cultivated in Indonesia coffee plantations and the green beans of these coffees are widely exported abroad as well. Ateng coffee is first known in Aceh Tengah, Gayo plateau, Aceh province, Sumatera. This variety resists pests and diseases, produces coffee cherries in a short time, 2 years after the cultivation, and has a high productivity [5]. Meanwhile, sigararutang is a superior variety of Arabica coffee that first developed in Lintong, Humbang Hasundutan, North Sumatera [6]. Sigararutang variety possesses several superior characteristics, including dwarf type (short), easily grown, rapid fruiting, dry resistance, high productivity, and very good flavor quality [7]. Based on the local farmer acknowledgment, buhun coffee variety originated from the wild arabica coffee surviving from the leaf rust disease in the Dutch colonial era. The word “buhun” originates from Sundanese meaning ancient or old. Thus, this coffee is possibly one of the oldest arabica coffee variety in Indonesia. Although many agricultural and biological information of these interesting arabica coffee varieties have been well known, however their chemical information is very limited in the literature.

Metabolomics is one of analytical methods that has been applied for studying the chemical profiles of arabica coffee varieties. NMR-based metabolomics, as one of the common techniques in metabolomics, had been employed successfully to classify green beans of arabica coffees based on their varieties [8, 9]. This method was also applied for revealing fingerprint of Brazilian arabica coffees [10]. MS-based metabolomics approach was also used to study green beans of arabica coffee varieties that are grown in the same edaphoclimatic conditions [11]. Furthermore, green beans of arabica coffee varieties had been evaluated by IR-based metabolomics as well [12, 13].

Some epidemiological studies reported that a moderate and prolonged coffee consumption could decrease type 2 diabetes risk [14, 15]. This beneficial effect was correlated with the capability of several coffee compounds to decrease the sugar level in the blood by inhibiting glucosidase enzymes [16, 17]. One of the most frequently targeted glucosidase enzymes for the antidiabetic assay is alpha-glucosidase. Inhibition of this enzyme could prevent the glucose formation obtained from the digestion of carbohydrates, resulting in decrease the risk of diabetic diseases.

In this report, ^1^H NMR-based metabolomics was applied to evaluate chemical profiles of the green beans of Indonesia arabica coffee varieties, including ateng, buhun, and sigararutang. These coffees were cultivated in the same geographic conditions. Principal component analysis (PCA), hierarchical clustering analysis (HCA), and partial least square discriminant analysis (PLSDA) were applied to evaluate the similarity and differences in the metabolite profiles of coffee samples. Chemical markers for each coffee variety were revealed by analyzing the S-plots generated from two-class OPLSDA (orthogonal projections to latent structure discriminant analysis) models. Furthermore, their bioactivities in inhibiting alpha-glucosidase enzyme were investigated as well. This report shed more light on the chemical information of arabica coffee varieties cultivated widely in Indonesia.

## 2. Materials and Methods

### 2.1. Chemicals

Deuterium oxide (D_2_O), 3-(trimethylsilyl)-2,2,3,3-tetradeuteropropionic acid sodium salt (TSP), KH_2_PO_4_, and K_2_HPO_4_ were bought from Merck (Darmstadt, Germany). Ascorbic acid, alpha-glucosidase from *Saccharomyces cerevisiae*, and p-nitrophenyl-alpha-D-glucopyranoside (PNPG) were purchased from Sigma-Aldrich (St. Louis, USA). Acarbose was obtained from TCI (Tokyo, Japan).

### 2.2. Samples

In this work, the green beans of 3 arabica coffee varieties, including ateng, buhun, and sigararutang, were kindly provided by Koperasi Produsen Kopi Mekar Tani Gunung Wayang. The seedlings of ateng and sigararutang coffees originated from Agriculture Department of Bandung Regency, Republic of Indonesia. Meanwhile, buhun coffee seedlings were acquired directly from the remaining coffee plants cultivated in the Dutch colonial era and located in Wayang mountain. These old mother coffee plants could survive from the leaf rust disease in the colonial era and are still found in some mountain areas of Bandung Regency. The coffee samples used in this research were cultivated in Wayang mountain area, (around 1600 m above sea level and average annual temperatures of 17-22°C), Kertasari, Bandung Regency, West Java, Indonesia. The green coffee beans used in this report were derived from the completely ripe coffee fruits that harvested in March to June 2018. The harvested fruits were processed by the wet method and then dried by the sun for obtaining the bean moisture of 11–12%. The green coffee beans were stored at -30°C until extraction. Ateng and buhun coffee samples consisted of 6 biological replicates each, while sigararutang sample composed of 5 biological replicates.

### 2.3. Sample Extraction

The green beans of the arabica coffees were ground with a 600 N coffee grinder (Yang Chia Machine Work, Taiwan) into powder. After that, 400 mg of the coffee powder was extracted with 2 mL of D_2_O (containing TSP 1 mM and sodium phosphate buffer, pH 6.00) in a plastic tube and then incubated in a hot water (95°C) for 30 minutes. The sample was chilled in the room temperature for 30 minutes and then centrifuged at 3000 rpm for 6 minutes with a Varispin 12R centrifuge (Cryste Separation Technology, Gyeonggi-do, South Korea). Finally, 500 *μ*L of the supernatant was transferred into a 5 mm NMR tube.

### 2.4. ^1^H NMR Spectroscopy

The spectra of ^1^H NMR were acquired with a Variant Unity INOVA-500 Spectrometer (Agilent Technologies, Santa Clara, United States) operating at 500 MHz. The ^1^H presaturation mode was applied to suppress the signal of H_2_O. For each ^1^H NMR spectrum, 128 scans were collected into 64 K data points using a spectral width of 8012.8 Hz with an acquisition time of 2.720 s and a delay time of 2.0 s. The Free Induction Decay (FID) files of the ^1^H NMR spectra were further processed with an ACD/Labs 12.0 software (ACD/Labs, Toronto, Canada). The baseline correction of each spectrum was performed. TSP (3-(trimethylsilyl)-2,2,3,3-tetradeuteropropionic acid sodium salt) signal was used for calibrating the chemical shift.

### 2.5. Quantitative Analysis

To quantify metabolites in the green coffee beans, the ^1^H NMR spectra were further analyzed based on a previous report [18] with slight modifications. The signal of TSP (1 mM) was applied as a quantitative reference signal since it has a singlet peak and does not overlap with other signals. The metabolite concentration was determined by comparing the proton signal integration of the targeted metabolites with the singlet signal integration of TSP. The statistical calculation of the quantitative analysis was carried out with Microsoft Excel 365 ProPlus.

### 2.6. Data Extraction

Alignment and bucketing of the ^1^H NMR spectra were performed with the ACD/Labs 12.0 software (ACD/Labs, Toronto, Canada). After the alignment, the spectra were bucketed into integrated bins with the equal width (0.04 ppm) within *δ* 0.50-10.00 ppm. This bucketing was carried out by activating the intelligent bucketing option. The buckets containing residual signals of water (*δ* 4.75-5.20 ppm) were removed from the analysis. Because of the alterations of the caffeine signals, the buckets at *δ* 3.22-3.49 ppm and *δ* 3.82-3.88 ppm were excluded as well. The extracted data were imported into a Microsoft Excel software and then normalized with the sum method to prevent the bias effects.

### 2.7. Multivariate Data Analysis

The normalized data were imported into SIMCA-P version 12.0 (Umetrics, Umeå, Sweden), and Pareto scaling was applied for the multivariate statistical analysis. For evaluating the intrinsic variation in the data, PCA (unsupervised approach) was applied in the beginning of the analysis. The 95% confidence interval of the model variation was explained by Hotelling's *T*^2^ technique revealed as an ellipse in the score plots. PLSDA and OPLSDA were employed as the main methods for discriminating the metabolomes of the coffee samples. The data of the green coffee beans were classified into 3 groups based on their varieties and then evaluated by PLSDA models. The quality of the models was depicted with *R*^2^*X*, *R*^2^*Y*, and *Q*^2^ values. The first two explain the variation in the data and exhibit the goodness of fit. Meanwhile, *Q*^2^ is defined as the variation predicted by the models based on cross validation. A permutation test with 200 iterations was performed for validating the PLSDA models. HCA was applied to evaluate the lineage relationship among samples. S-plots created from two-class OPLSDA models were analyzed for revealing the discriminant metabolites for each coffee variety.

### 2.8. Alpha-Glucosidase Inhibition Assay

Alpha-glucosidase inhibition assay was performed based on a reported work [19] with slight modifications. This assay was conducted in the 96-well microplates with a xMark™ microplate absorbance spectrophotometer (Bio-Rad, California, USA). About 5 grams of the powder of green coffee beans was extracted with 40 mL of milli-Q deionized water (1 : 8, w/v). The supernatant was filtered and then dried in a freeze dryer machine (Operon, Gyeonggi-do, South Korea). The dried extract was dissolved in the deionized water: methanol (9 : 1, v/v). 3.5 *μ*L of the sample was mixed with 200 *μ*L of phosphate buffer pH 7.0 containing 10 *μ*L of alpha-glucosidase 1 U/mL and then incubated for 10 minutes at 37°C. It was mixed with 35 *μ*L of *p*-nitrophenyl-*α*-D-glucopyranoside (PNPG) 5 mM (dissolved in phosphate buffer pH 7.0), incubated for 15 minutes at 37°C, and then measured with the UV spectrometer at the wavelength of 405 nm. The sample without the coffee extract was used as the control group; meanwhile, the sample without alpha-glucosidase enzyme was used as the blank. In this assay, the concentration of the sample was varied within the range of 0.25-2.00 mg/mL. IC_50_ value was determined by the linear regression between the inhibition percentage and the sample concentration.

## 3. Results and Discussion

### 3.1. Metabolite Identification

**Figure 1:**
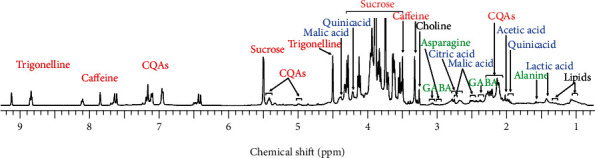
Signal assignments of the identified metabolites in the ^1^H NMR spectrum of the green coffee beans.

**Figure 2:**
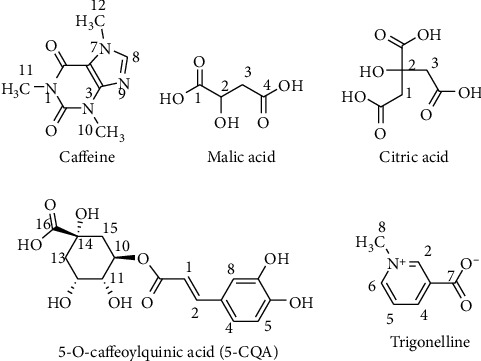
Some molecular structures of the identified compounds in the green coffee beans.

In this work, metabolites in the green coffee beans were identified by detecting the fingerprint signals in the obtained ^1^H NMR spectra. In total, 18 metabolites had been successfully detected in the ^1^H NMR spectra. The signals belong to caffeine, sucrose, trigonelline, and chlorogenic acids (3-caffeoyl quinic acid (3-CQA), 4-caffeoyl quinic acid (4-CQA), and 5-caffeoyl quinic acid (5-CQA)) were clearly detected in the spectra as depicted in Figure 1. Thus, it indicated that those were major metabolites in the green coffee beans. Other acidic compounds were also identified, including acetic acid, citric acid, formic acid, lactic acid, malic acid, and quinic acid. Three amino acids were detected in the spectra as well, namely, alanine, asparagine, and gamma-aminobutyric acid (GABA). Other identified compounds in the green coffee bean samples were choline, myo-inositol, and lipids. The fingerprint signals of the detected compounds are described in Table 1. The fingerprint signals of the identified metabolites were farther confirmed with 2D NMR analysis, including J-resolved, COSY, and TOCSY techniques. Moreover, the assigned signals were further verified with their corresponding reference spectra obtained from the HMDB database (https://hmdb.ca/) and with NMR data of the green coffee beans reported in the literature [8, 9, 20]. Some structures of the identified metabolites are described in Figure 2.

### 3.2. Metabolite Quantification

Concentrations of some identified metabolites in the coffee samples were further determined semiquantitatively with the ^1^H NMR technique. Nonoverlapped signals of the quantified metabolites were chosen for the quantification. The amounts of 5-CQA, alanine, acetic acid, caffeine, GABA, choline, sucrose, and trigonelline were successfully calculated as shown in Table 2. Concentrations of major metabolites, including 5-CQA, caffeine, sucrose, and trigonelline, were higher in the green beans of ateng coffee than in the other arabica coffee samples. For instance, the concentrations of trigonelline in the samples of ateng, sigararutang, and buhun were 10.54 ± 0.26, 8.82 ± 1.26, and 6.83 ± 0.58 mM, respectively. Meanwhile, minor metabolites found in the highest amount in the green beans of this coffee variety were alanine and acetic acid. The concentration of acetic acid in the coffee samples of ateng, sigararutang, and buhun was 3.43 ± 0.18, 1.46 ± 0.05, and 2.28 ± 0.25 mM, respectively.

As depicted in Table 2, the lowest amounts of the quantified metabolites, including 5-CQA, alanine, acetic acid, caffeine, sucrose, and trigonelline, were found mostly in sigararutang coffee samples. However, the green beans of this coffee variety possessed the highest concentration of GABA. For instance, the levels of GABA in buhun, ateng, and sigararutang coffees were 1.75 ± 0.01, 1.87 ± 0.08, and 2.35 ± 0.19 mM, respectively. Meanwhile, the concentrations of choline in all coffee samples did not differ significantly as explained by the *P* value of ANOVA analysis (Table 2).

### 3.3. Classification of the Green Beans of Arabica Coffees

**Figure 3:**
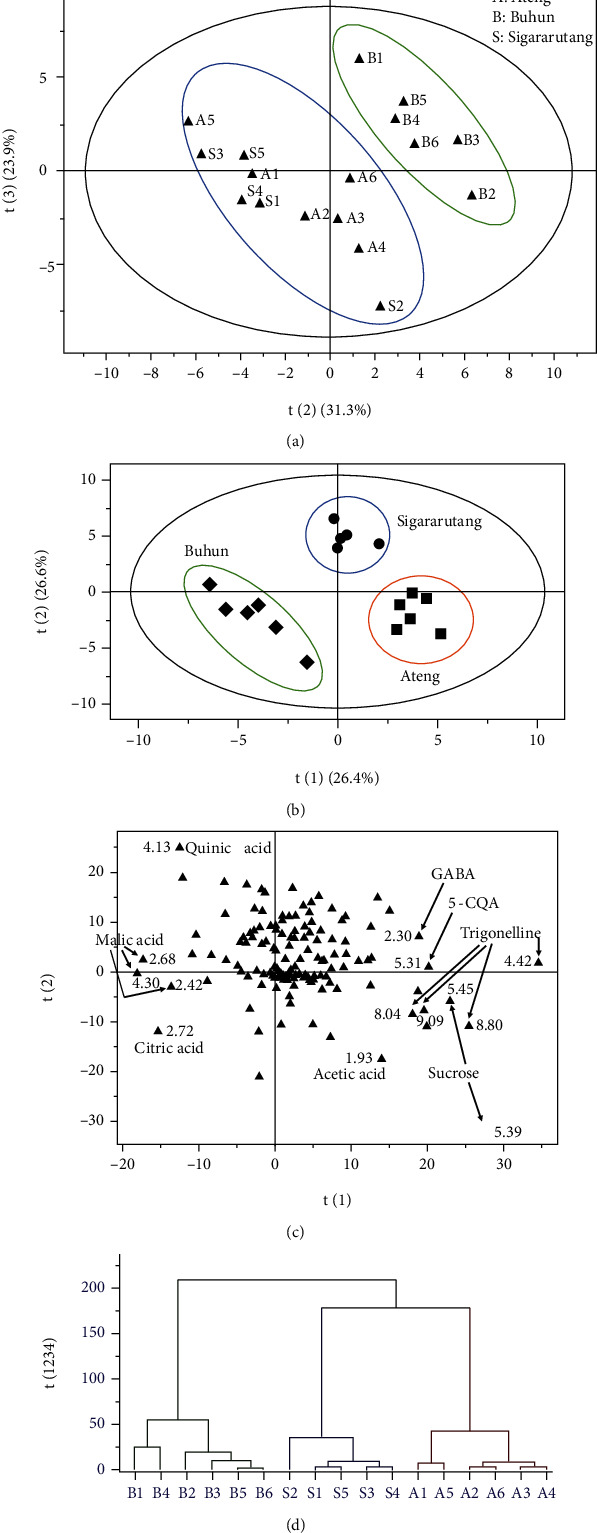
Multivariate data analysis computed for the green beans of the 3 Indonesian arabica coffee varieties. (a) PCA score plot separated buhun coffee from the others. (b) PLSDA score plot successfully classified the coffee samples based on their varieties. (c) PLSDA loading plot revealed the important buckets contributing to the classification. (d) HCA plot showed the lineage relationship within the coffee samples. A: ateng; B: buhun; S: sigararutang.

The data set extracted from the ^1^H NMR spectra was evaluated with multivariate data analysis for classifying the green coffee beans according to their varieties. In the beginning of the analysis, principal component analysis (PCA, unsupervised approach) was applied. The PCA model of the green coffee beans comprised 4 principal components explaining 82.2% of total variation (*R*^2^*X*) with predictive ability of 46.0% (*Q*^2^). The best group separation on score plot of the PCA model was obtained by combining PC 2 (31.3%) and PC 3 (23.9%) as depicted in Figure 3(a). Although this score plot could not distinguish properly ateng and sigararutang coffees, however it successfully separated buhun coffee from the others. Moreover, this score plot indicated that ateng and sigararutang had more similarity in the metabolite profiles.

The PLSDA model of the green beans of the arabica coffees was generated for obtaining the better group classification. This PLSDA model consisted of 4 components defining 79.3% and 95.5% of the total variations (*R*^2^*X* and *R*^2^*Y*, respectively) and 74.5% of cross validation coefficient (*Q*^2^). This model was validated by conducting 200 rounds of a random permutation of the *Y* variable resulting the regressions of *Q*^2^ lines intersected the *y*-axis at points below zero (*Q*^2^ = (0.00, −0.502); *R*^2^ = (0.00, 0.507)). Combination of the first (26.4%) and the second (26.6%) PLSDA components resulted the score plot that could classify the green beans of the arabica coffees based on their varieties as shown in Figure 3(b). The corresponding loading plot was analyzed to reveal the buckets involved in the classification. The buckets belong to trigonelline, sucrose, 5-CQA, GABA, malic acid, citric acid, quinic acid, and acetic acid were found contributing to the classification of the green coffee beans based on the varieties as seen in Figure 3(c).

In order to reveal the cluster hierarchy of arabica coffee samples, the data set was evaluated with HCA. As depicted in Figure 3(d), HCA clearly classified the green bean metabolomes according to their coffee varieties. Furthermore, HCA showed that sigararutang and ateng samples were in the same cluster line; meanwhile, buhun coffee was in the different cluster line from the other samples. Therefore, based on the metabolome profiles in the HCA, sigararutang and ateng coffees possessed a closer relationship compared with buhun coffee.

Ateng coffee is a catimor type, derived from a natural hybrid between timor and caturra varieties [21]. Timor variety is a natural cross between *C. arabica* var. typica and *C. canephora* (robusta), while caturra is a natural mutation of *C. arabica* var. borboun. Sigararutang variety is considered as a result of natural cross breeding between blawan pasumah (derived from typica) and catimor [6]. Thus, both had the catimor lineage and indicated having a close lineage relationship. Interestingly, this was confirmed by the previous HCA analysis describing both in the same cluster line. Besides having arabica coffee characteristics, ateng and sigararutang inherit some robusta coffee properties as well. Meanwhile, buhun only possesses arabica coffee characteristics since it is derived from *C. arabica* var. typica [22]. Therefore, the lineage of buhun coffee is weakly correlated with ateng and sigararutang coffees via the typica variety connection. It was verified by the HCA plot showing buhun coffee was in the different cluster line from ateng and sigararutang coffees. Interestingly, this result was in accordance with their genetic relationship reported in the previous work [22].

**Figure 4:**
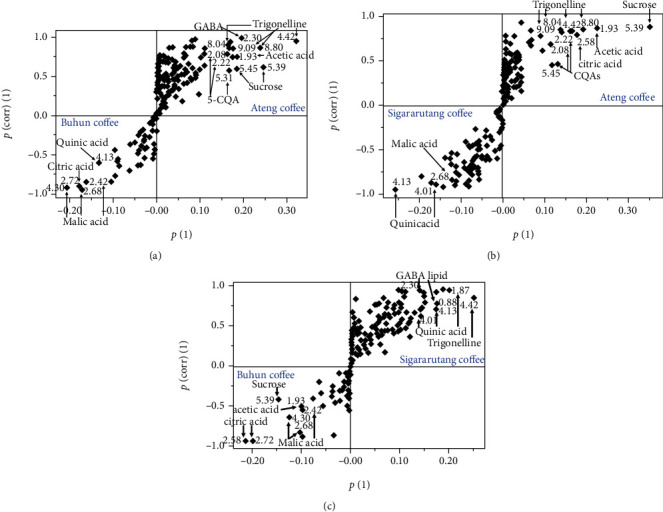
S-plots of two-class OPLSDA models of the green beans of the three Indonesian arabica coffee varieties: (a) ateng and buhun, (b) ateng and sigararutang, and (c) buhun and sigararutang.

To obtain a better analysis of the characteristic metabolites for each variety, two-class OPLSDA models were created. In total, 3 two-class OPLSDA models were successfully generated. S-plots of these OPLSDA models were further investigated to acquire the metabolites influencing the coffee discriminations. The first S-plot was derived from the OPLSDA model of ateng and buhun (*R*^2^*X* = 82.9%; *R*^2^*Y* = 99.7%; *Q*^2^ = 96.1%). As depicted in Figure 4(a), trigonelline (buckets at 4.42, 8.04, 8.80, and 9.09 ppm), sucrose (buckets at 5.39 and 5.45 ppm), 5-CQA (buckets at 2.08, 2.22, and 5.31 ppm), GABA (bucket at 2.30 ppm) and acetic acid (bucket at 1.93 ppm) were found as characteristic compounds of ateng coffee. Meanwhile, buhun coffee was characterized with malic acid (buckets at 2.42, 2.68, and 4.30 ppm), citric acid (bucket at 2.72 ppm), and quinic acid (bucket at 4.13 ppm).

The second S-plot was obtained from the two-class OPLSDA model of ateng and sigararutang (*R*^2^*X* = 82.6%; *R*^2^*Y* = 99.8%; *Q*^2^ = 86.3%). Interestingly, most metabolites found as characteristic compounds of ateng coffee in the previous S-plot, including sucrose, trigonelline, 5-CQA, and acetic acid, together with citric acid were discovered as the discriminant metabolites in this S-plot (Figure 4(b)). These results were in accordance with the quantitative analysis exhibiting the highest concentrations of sucrose, trigonelline, 5-CQA, and acetic acid were found in the green beans of ateng coffee (Table 2). Meantime, sigararutang coffee was identified by quinic acid (buckets at 4.13 and 4.01 ppm) and malic acid (bucket at 2.68 ppm).

The two-class OPLSDA model of buhun and sigararutang (*R*^2^*X* = 86.2%; *R*^2^*Y* = 99.6%; *Q*^2^ = 94.1%) resulted the last S-plot. As described in Figure 4(c), quinic acid (buckets at 1.87, 4.01, and 4.13 ppm), trigonelline (bucket at 4.42 ppm), GABA (bucket at 2.30), and lipid (bucket at 0.88 ppm) were observed as discriminant compounds of sigararutang coffee in this S-plot. Among these metabolites, quinic acid was the only metabolite found as the discriminant compound of sigararutang coffee in each corresponding S-plot, indicating as the important marker for this coffee variety. Meanwhile, the last S-plot also revealed that malic acid (buckets at 2.68 and 4.30 ppm), citric acid (buckets at 2.58 and 2.72 ppm), acetic acid (bucket at 1.93 ppm), and sucrose (bucket at 5.39 ppm) were discriminant compounds of buhun coffee. The first two compounds were consistently found as the discriminant compounds of this sample, indicating both were the important characteristic compounds for buhun coffee.

### 3.4. Alpha-Glucosidase Inhibitory Activity

In this work, the inhibitory activity of the green beans of arabica coffee samples against alpha-glucosidase enzyme was evaluated. The results showed that the inhibitory activity of the coffee samples did not vary widely (Table 3). Moreover, ateng and sigararutang coffees possessed similar IC_50_ values of the alpha-glucosidase inhibitory activity, which are 3.01 ± 0.16 mg/mL and 3.14 ± 0.20 mg/mL, respectively. The similarity of the alpha-glucosidase inhibitory activity between ateng and sigararutang possibly was caused by the resemblance of their metabolite profiles as explained by HCA and PCA models before. Meanwhile, green beans of buhun coffee inhibited alpha-glucosidase with IC_50_ value of 5.05 ± 0.28 mg/mL indicating the lowest activity among the tested samples.

Among the discriminant compounds of each coffee sample explained before, only 5-CQA, citric acid, malic acid, and trigonelline had been reported having alpha-glucosidase inhibitory activity [15, 16, 23, 24]. Moreover, it had been reported that the content of malic acid possibly possessed a positive correlation with alpha-glucosidase inhibitory activity of Luwak coffees [25]. Therefore, these compounds possibly contributed to the alpha-glucosidase inhibitory activity of the green coffee bean samples.

The correlation of alpha-glucosidase inhibitory activity with the concentration of some quantified compounds, including 5-CQA, caffeine, and trigonelline, was investigated with the Pearson correlation coefficient test. The results revealed that the levels of 5-CQA (*r* = 0.445, *P* value = 0.707) and caffeine (*r* = 0.689, *P* value = 0.516) insignificantly had positive relationships with the IC_50_ values of the alpha-glucosidase inhibitory activity. Meanwhile, concentration of trigonelline significantly possessed a positive correlation with alpha-glucosidase inhibitory activity of the coffee samples (*r* = 0.998, *P* value = 0.045). Therefore, this result supported that trigonelline contributed to the alpha-glucosidase inhibitory activity of the coffee samples.

## 4. Conclusions

Metabolite profiles of the green beans of 3 Indonesian arabica coffee varieties, including ateng, buhun, and sigararutang, had been evaluated successfully with ^1^H NMR-based metabolomics. Multivariate data analysis using PCA and HCA models revealed the lineage relationship within these coffee varieties. Ateng and sigararutang coffees, having catimor lineage, possessed the resemblant metabolite profiles. Both are the most cultivated arabica coffee varieties in Indonesia because of their superior properties. The metabolite profile of buhun coffee, possibly one of the oldest coffee varieties in Indonesia, had a unique metabolite profile and different from the others. It was possibly caused by the fact that buhun coffee is a pure arabica coffee derived from the variety of typica, while ateng and sigararutang coffees possess not only arabica coffee lineage but also robusta coffee originated from timor variety. However, the metabolite profiles of all coffee varieties were successfully discriminated by PLSDA and the two-class-OPLSDA models. Sucrose, trigonelline, 5-CQA, quinic acid, acetic acid, citric acid, malic acid, and GABA were determined as biomarkers responsible for discriminating the metabolite profiles. The results of our work provided meaningful data for Indonesian arabica coffee studies and scientific information for developing the superior coffee varieties. Moreover, our study confirmed ^1^H NMR-based metabolomics as a reliable method for revealing the lineage relationship in the samples.

## Figures and Tables

**Table 1:** Characteristic ^1^H NMR signals of the identified compounds.

No.	Compound	Chemical shift (ppm)
1.	3-CQA	2.05 (H-13a and H-15a, m), 2.20 (H-13e and H-15e, m), 6.39 (H-1, d), 6.86 (H-5, d), 7.03 (H-4, m), 7.12 (H-8, brs)7.60 (H-2, d)
2.	4-CQA	2.05 (H-13a and H-15a, m), 2.20 (H-13e and H-15e, m), 4.34 (H-12, m), 6.36 (H-1, d), 6.86 (H-5, d), 7.02 (H-4, m), 7.09 (H-8, brs), 7.57 (H-2, d)
3.	5-CQA	2.05 (H-13a and H-15a, m), 2.20 (H-13e and H-15e, m), 3.90 (H-11, dd), 4.27 (H-12, brs), 5.34 (H-10, m), 6.31 (H-1, d), 6.86 (H-5, d), 7.00 (H-4, m), 7.06 (H-8, brs), 7.54 (H-2, d)
4.	Acetic acid	1.93 (H-2, s)
5.	Alanine	1.49 (H-3, d)
6.	Asparagine	2.87 (H-3b, dd), 2.97 (H-3a, dd)
7.	Caffeine	3.22 (H-11, s), 3.39 (H-10, s), 3.85 (H-12, s), 7.77(H-8, s)
8.	Choline	3.21 (H-3, H-4, H-5, s), 3.49 (H-1, t)
9.	Citric acid	2.62 (H-1, d), 2.72 (H-3, d)
10.	Formic acid	8.47 (s)
11.	GABA	2.32 (H-4, t), 3.03 (H-2, t)
12.	Myo-inositol	3.27 (H-5, t), 3.53 (H-2, m), 3.62 (H-4, H-6, m)
13.	Lactic acid	1.34 (H-3, brs), 4.12 (H-2, m)
14.	Lipid	0.90 (m), 0.96 (brs), 1.30 (m)
15.	Malic acid	2.42 (H-2a, m), 2.68 (H-2b, m), 4.33 (H-1, m)
16.	Quinic acid	1.89 (H-2a, dd), 1.98 (H-6a, m), 2.05 (H-2e and H-6e, m), 4.01 (H-3, m), 4.16 (H-5, m)
17.	Sucrose	3.46 (H-4, t), 3.57 (H-2, dd), 3.69 (H-1′, s), 3.78 (H-3, t), 3.84 (H-6, H-6′, m), 3.86 (H-5, m), 3.90(H-5′, m), 4.07 (H-3′, t), 4.23 (H-4′, d), 5.42 (H-1, d)
18.	Trigonelline	4.43 (H-8, s), 8.07 (H-5, t), 8.82 (H-6, m), 8.84 (H-4, m), 9.11 (H-2, s)

**Table 2:** Concentrations of some identified metabolites determined by quantitative ^1^H NMR analysis.

No.	Compound	Metabolite concentration in green coffee beans (mM)			
		Ateng (±SD)	Buhun (±SD)	Sigararutang (±SD)	*P* value
1.	5-CQA (*δ* 6.26–6.35 ppm)	17.34 (±1.07)	11.55 (±1.10)	10.87 (±1.18)	5.79*E*-04
2.	Acetic acid (*δ* 1.93–1.96 ppm)	3.43 (±0.18)	2.28 (±0.25)	1.46 (±0.05)	3.76*E*-05
3.	Alanine (*δ* 1.47–1.25 ppm)	1.92 (±0.03)	1.38 (±0.10)	1.28 (±0.09)	1.69*E*-04
4.	Caffeine (*δ* 7.75–7.80 ppm)	9.42 (±0.22)	7.09 (±0.46)	6.01 (±0.66)	3.62*E*-04
5.	Choline (*δ* 3.19–3.21 ppm)	1.02 (±0.07)	0.93 (±0.10)	1.08 (±0.09)	0.21
6.	GABA (*δ* 3.00–3.05 ppm)	1.87 (±0.08)	1.75 (±0.01)	2.35 (±0.19)	0.002
7.	Sucrose (*δ* 5.39–5.45 ppm)	55.58 (±2.51)	36.09 (±3.22)	31.56 (±3.73)	2.06*E*-04
8.	Trigonelline (*δ* 8.45–8.49 ppm)	10.54 (±0.26)	6.83 (±0.58)	8.82 (±1.26)	5.84*E*-05

*P* values are derived from one-way ANOVA. *P* values < 0.05 mean significantly different.

**Table 3:** IC_50_ values of alpha-glucosidase inhibitory activity of the coffee samples.

No.	Samples	IC_50_ (mg/mL) ± SD
1.	Green beans of ateng coffee	3.01 ± 0.16
2.	Green beans of buhun coffee	5.05 ± 0.28
3.	Green beans of sigararutang coffee	3.14 ± 0.20

## Data Availability

All the data relevant to the research can be found in the manuscript. Further information is available from the corresponding author upon the request.
